# The effect of live body condition score of beef cows on carcass characteristics, carcass-cutting yields, processor profitability, and tenderness in the *longissimus lumborum* and *psoas major* muscles

**DOI:** 10.1093/tas/txae129

**Published:** 2024-08-27

**Authors:** Kayla G Scott, Yifei Wang, Benjamin M Bohrer, Lyda G Garcia

**Affiliations:** Department of Animal Sciences, The Ohio State University, Columbus OH 43210, USA; Department of Animal Sciences, The Ohio State University, Columbus OH 43210, USA; Department of Animal Sciences, The Ohio State University, Columbus OH 43210, USA; Department of Animal Sciences, The Ohio State University, Columbus OH 43210, USA

**Keywords:** beef cows, beef quality, beef sustainability, cull cows, re-alimentation period

## Abstract

The objective of this study was to investigate the effects of body condition score (BCS) of cull cows on carcass characteristics, carcass-cutting yields, profitability, and tenderness development for the *longissimus lumborum* and *psoas major* muscles. During a 5-wk period (May–June 2023), 10 boner cull cows (BCS 4 to 6) and 10 leaner cull cows (BCS 1 to 3) were purchased at a commercial auction market. Live conformation, carcass characteristics, weights of fabricated cuts, tenderness, pH decline, and temperature decline were recorded following slaughter. Carcasses were fabricated into the following cuts: knuckle, inside round, outside round, eye of round, strip loin, top sirloin, bottom sirloin flap, tenderloin, flank, ribeye roll, chuck tender, and brisket, whereas lean was separated into lean and fat components. Beef processor returns for boner cows were calculated as the sum of USDA Carlot Report values for the subprimal cuts, trim, bone, and drop value subtracted by actual live costs at the auction market whereas beef processor returns for leaner cows were calculated as the sum of USDA Carlot Report values for the trim, bone, and drop value subtracted by actual live costs at the auction market. Carcass and merchandizing value parameters were analyzed using a completely randomized design with a fixed effect of classification (leaner or boner) and a random effect of slaughter day. Live weight was used as a covariant for all carcass and merchandizing value parameters due to the pronounced effect of live weight for most parameters. Temperature decline, pH decline, shear force, and cooking loss parameters were analyzed using a completely randomized design with a fixed effect of classification (leaner or boner) and a random effect of slaughter day. Boner cows were found to be heavier for both live and carcass weights when compared with leaner cows. In addition, backfat thickness was 0.39 cm greater (*P* = 0.03), and ribeye area was 9.17 cm^2^ (*P* = 0.05) greater for boner cows compared with leaner cows. This resulted in boner cows yielding larger subprimal cuts and a greater amount of lean trim, which in turn generated more beef processor revenue. However, specific consideration should be provided for profitability as the ability to market subprimal cuts is highly dependent on muscle size, fat deposition, meat quality, and market prices for both boner and leaner cows.

## Teaser

Beef cull cows representing thin body condition scores will result in lower percentage yields due to less saleable products when compared with beef cull cows representing average body condition scores. Therefore, consideration for meat quality and merchandizing value should be provided when purchasing cull cows of differing levels of body condition score.

## Introduction

In the United States, the beef cow herd has experienced annual reductions in size since 2019 (reaching historical lows of 28.9 million animals in 2023), which the United States Department of Agriculture (USDA)-ERS attributes to the biological nature of the beef cattle business and responses to changes in market prices and climate conditions (USDA-ERS, 2023). Annual culling rates for beef cows in the United States can reach up to 10% to 15% of the total beef cow herd and can be influenced by the aforementioned market prices and climate conditions, particularly in times of high beef demand and/or in times of low forage availability. Tangibly, beef cows are culled for a variety of reasons with the 2 most common reasons being due to pregnancy status (e.g., open or not pregnant) and age (Peel, 2022). A cow’s ability to achieve and maintain a pregnancy is the most important production factor as it relates to profitability because weaned calves are the main source of income for a cow–calf operation. Drought conditions, and their inherent effect on forage availability and in turn nutrition of cows, have a significant influence on reproductive performance (Diskin and Kenny, 2016; Rodziewicz et al., 2023). In addition, age, and specifically poor condition of teeth, can hinder a cow’s ability to consume adequate levels of nutrition. Cows with poor dental health struggle to meet their nutritional requirements and as a result will have a decline in body weight and overall body condition (i.e., low levels of fat deposition; Hersom et al., 2016). Regardless of the cause, failure to meet nutritional requirements and poor body condition can be associated with low levels of reproductive success (Renquist et al., 2006; Kunkle et al., 2021). Body condition score (BCS) is used to estimate the relative fatness (or lack of) for beef and dairy cows. A scoring system of 1 to 9 is used for beef cull cows. A thin BCS typically ranges from 1 to 3; an average BCS typically ranges from 4 to 6; and an obese BCS typically ranges from 7 to 9. Beef cull cows are typically categorized into 4 classes by the beef processing industry based on BCS: 1) breakers; 2) boners; 3) leaners; and 4) lights. Breakers are the fattest and typically align with BCS 7 or greater, boners are the next category and typically align with BCS 4 to 6, leaners and lights are the next 2 categories and typically align with BCS 1 to 3 (lights are further defined as cows that are also light weight, specifically with carcass weights below 227 kg) (Selk, 2008). Cows representing average BCS (4 to 6) have previously been found to have the highest percentage of lean yields from primals and subprimals (Apple et al., 1999). Greater initial body weights have been shown to have positive impacts on hot carcass weight, area of the *longissimus* muscle as well as having an increase in USDA yield grade (Funston et al., 2003). The official summary from the most recent National Beef Quality Audit (NBQA) highlighted an increase in the proportion of light-muscled cull cows or cows labeled as “too thin” compared to prior audits; specifically, the percentage of cattle labeled with a BCS of 1 or 2 increased by nearly 13% over a 5-yr period (Harris et al., 2017; Borders et al., 2024). As cows of average BCS (4 to 6) have been reported to have the highest-yielding carcasses in previous studies, it is equally important to assess processor profitability and meat quality.

There is a significant need to benchmark meat-cutting yields and meat quality traits of different classifications of cull cows. Within the industry, we believe beef processors have realized that some market cows have the potential to yield valuable retail cuts, particularly boneless cuts originating from the rib, loin, and sirloin primals. Furthermore, we hypothesize that processing profitability and meat quality will be improved when cows have more muscling and finish. In order to test this hypothesis, the objective was to determine the effect of BCS in cull cows on carcass characteristics, carcass-cutting yields, processing profitability, and development of tenderness for the *longissimus lumborum* and *psoas major* muscles.

## Materials and Methods

### Animals and Slaughter Procedures

A total of 20 *Bos taurus* cows comprised of beef (i.e., nondairy breed) genetics were procured from a commercial auction market over a 5-wk period spanning from May to June 2023. Ten cows with BCS from 1 to 3 were utilized as the leaner classification group and 10 cows with BCS from 4 to 6 represented the boner classification group. All cows were assessed and scored by a single commercial cattle buyer (>50 yr of experience) using the 9-point body condition scoring system (Nicholson, 2010). This scoring system was utilized previously by National Cattlemen’s Beef Association during the NBQA (Harris et al., 2017; Borders et al., 2024). Cows were weighed at the time of purchase from the commercial auction market and again prior to slaughter at the Ohio State University meat science laboratory. Shrinkage (drift) between the 2 locations was calculated as the difference between the 2 live weights divided by the weight at the time of purchase from the commercial auction market and expressed as a percentage. Cows were slaughtered on 3 slaughter dates (over a 5-wk period) under federal inspection using approved humane slaughter procedures. Each slaughter date consisted of 6 to 7 cows (3 to 4 cows representing each classification).

Udder weight was collected during slaughter. Carcasses were weighed (hot carcass weight) immediately following final dressing and federal inspection on the slaughter floor. Dressing percentage was calculated as hot carcass weight divided by live weight and expressed as a percentage. Muscling score was also recorded using the muscling score system reported by Nicholson (2010) which was used in the 2007 National Cow and Bull Quality Audit and subsequent NBQA.

### Carcass Evaluation Factors

At f48-h postmortem, carcasses were evaluated for traits related to yield grade and quality grade. Yield grade traits included backfat thickness at the 12th/13th rib interface, ribeye area at the 12th/13th rib interface, an estimation of kidney pelvic and heart (KPH) fat, and hot carcass weight. Quality grade traits included marbling score at the 12th/13th rib interface, skeletal maturity along the split line, dentition, and lean maturity at the 12th/13th rib interface. In addition, muscle score, fat color, and bruises were recorded. Data were collected by 2 trained Ohio State personnel who utilized a USDA ruler, a beef ribeye dot grid, and USDA marbling cards as points of reference. Muscle score and fat colors were determined by comparing the carcass to the scoring system provided by the National Cow and Bull Quality Audit (Nicholson, 2010). Finally, each carcass was observed for carcass defects, specifically dark cutter lean color, blood splash, and bruising (recorded as number and size per side).

### Carcass Cutting Tests

Carcass-cutting tests were conducted 96 h postmortem. Left sides of carcasses were individually weighed (cold carcass weight) prior to fabrication and then fabricated into primals and subprimals according to Institutional Meat Purchase Specifications (IMPS) (NAMP, 2006; IMPS, 2014). Weights (kg) were collected on the following subprimals: knuckle [IMPS# 167A; beef round, sirloin tip (knuckle), peeled], inside round [IMPS# 169A; beef round, top (inside), cap off], outside round [IMPS# 171B; beef round, outside round (flat)], eye of round (IMPS# 171C; beef round, eye of round), striploin (IMPS# 180; beef loin, strip loin, boneless), top sirloin (IMPS# 184; beef loin, top sirloin butt, boneless), bottom sirloin flap (IMPS #185A; beef loin, bottom sirloin butt, flap, boneless), tenderloin (IMPS #189; beef loin, tenderloin, full), flank (IMPS #193; beef flank, flank steak), ribeye roll (IMPS #112 beef rib, ribeye roll), chuck tender (IMPS #116A, beef chuck, chuck tender), and brisket (IMPS #120; beef brisket, deckle-off, boneless). Additionally, weights for trim, fat, and bone were recorded.

### Merchandized Cut Value

Current market value (as of June 23, 2023; USDA Carlot Report, 2023) was assigned for each of the subrimals that were fabricated and for trim, fat, and bone components for each carcass. In addition, drop value (i.e., hide and offal) was estimated based on the USDA Carlot Report for cull cows (USDA Carlot Report, 2023). Value of each of the cull cows was estimated using the following equations:

#### Total value of merchandised product


$$\begin{array}{llll} Boner\;cows\; & (when\;merchandized\;as\;subprimal\;cuts\;+trim+bone)\; & =\;total\;value\;of\;merchandized\;cuts\;+\;lean\;trim\;value\;\; & \;+\;fat\;trim\;value\;+\;bone\;value. \end{array}$$

$$\begin{array}{llll} Leaner\;cows\; & \left(when\;merchandized\;as\;trim+bone\right)\; & =\;lean\;trim\;value\;(assuming\;all\;subprimal\;cuts\;are\;\; & \;sold\;at\;lean\;trim\;value)+fat\;trim\;value+bone\;value. \end{array}$$

### Sample Collection

Muscle pH and temperature of the *longissimus lumborum* and *psoas major* muscles from the right side of each carcass were monitored at 2-, 4-, 8-, 24-, 48-, 72-, and 96-h postmortem. Temperature was measured using a digital meat thermometer probe (Taylor USA; Oak Brook, IL, USA) and muscle pH was measured using a calibrated MPI pH meter (Meat Probes Inc.; Topeka, KS, USA).

Following carcass-cutting tests, 2.5-cm thick steaks consisting of the *longissimus lumborum* and *psoas major* muscles were collected from the left sides of the carcasses and vacuum-packaged. Steaks were randomly assigned to postmortem aging periods of 4, 7, 14, 21, 28, 35, or 42 d. Steaks were frozen at −20°C once the aging times elapsed.

### Laboratory Assays

Cooking loss was calculated for each muscle sample by first weighing the samples while still in each individual vacuum-sealed bag. Each sample was cooked in a water bath to an internal temperature of 71°C and allowed to rest for at least 2 h in a refrigerated room set to 4°C. Each sample was removed from its bag and weighed with its original identification tag to calculate cooking loss. Round cores running parallel to the muscle fibers were collected from each sample using a 1.3-cm diameter coring drill bit. Cores were analyzed for instrumental tenderness with a texture analyzer (TA.XTplusC; Texture Technologies, Hamilton, MA, USA) using Warner–Bratzler shear force procedures. Test parameters included a pretest and test speed of 2.0 mm/s with the peak force for each core captured and then averaged across the cores from a given muscle sample.

### Statistical Analysis

Carcass and merchandizing value parameters were analyzed using a completely randomized design with a fixed effect of classification (leaner or boner) and a random effect of slaughter day. Live weight at the Ohio State University meat lab was used as a covariant for all carcass and merchandizing value parameters due to the significant (and pronounced) effect of live weight for most parameters. Temperature decline, pH decline, shear force, and cooking loss parameters were analyzed using a completely randomized design with a fixed effect of classification (leaner or boner) and a random effect of slaughter day. PROC GLIMMIX of SAS (SAS v. 9.4, Cary, NC) was used for means separation, and least squares means were separated using the PDIFF option of SAS, and a Tukey–Kramer adjustment was used to protect against committing a type-I statistical error. A *P*-value of 0.05 was used to determine significance.

## Results

### Live Evaluation and Carcass Characteristics

Cull cow BCS was 1.21 units greater (*P* < 0.01) and live muscling score was 0.96 units greater (*P *< 0.01) for boner cows compared with leaner cows (Table 1). There was a numerical difference in animal live weight at both the auction market and at the university meat lab of 50 kg; however, this value was not different (*P* = 0.14) between the boner and leaner cows. Because of the large numerical difference of live weight attributed to classification, live weight measured at the university meat lab was used as a covariate for carcass and merchandizing value parameters. Shrinkage from the auction market to the university meat lab was not different (*P* = 0.99) between boner and leaner cows.

**Table 1.** A comparison of live and carcass characteristics between boner and leaner beef cull cows

Variable	Boner	Leaner	Effect (Boner—Leaner)	95% CI	SED	Covariate *P* value	*P* value
Observations, *n*	10	10					
Body condition score^1^	4.04	2.83	1.21	(0.56, 1.85)	0.30	0.30	<0.01
Live muscling score^2^	2.88	1.92	0.96	(0.37, 1.54)	0.27	0.59	<0.01
Live weight at auction market, kg	556.00	506.00	50.00	(−18, 118)	32.00	—	0.14
Live weight at university meat lab, kg^3^	531.00	481.00	50.00	(−18, 119)	32.00	—	0.14
Shrink difference between auction market and university meat lab, %	5.50	5.46	0.04	(−6.53, 6.62)	3.07	0.15	0.99
Hot carcass weight, kg	270.60	250.50	20.10	(0.1, 40.2)	9.40	<0.01	0.05
Dressing percentage, %	53.39	49.45	3.94	(0.05, 7.84)	1.83	0.65	0.05
Udder weight, kg	6.46	6.00	0.46	(−2.77, 3.69)	1.52	0.41	0.77
Cold carcass weight, kg	263.60	243.20	20.40	(0.3, 40.4)	9.40	<0.01	0.05
Cooler shrink, %	2.54	2.98	−0.44	(1.25, 0.36)	0.38	0.95	0.26
Backfat thickness, cm	0.58	0.19	0.39	(0.05, 0.72)	0.16	0.11	0.03
Ribeye area, cm^2^	58.24	49.07	9.17	(0.08, 18.25)	4.26	0.01	0.05
Kidney, pelvic, and heart fat, %	0.92	0.43	0.49	(−0.05, 1.04)	0.26	0.08	0.07
Yield grade	2.59	2.48	0.11	(−0.53, 0.75)	0.30	0.15	0.72
Marbling score^4^	444	313	131	(2, 261)	61	0.58	0.05
Skeletal maturity^5^	466	588	−122	(−222, −21)	47	0.95	0.02
Lean maturity^5^	378	419	−41	(−115, 33)	35	0.49	0.26
Number of bruises	1.68	1.22	0.46	(−0.67, 1.60)	0.53	0.83	0.40
Carcass muscle score^6^	6.00	3.80	2.20	(0.8, 3.6)	0.70	0.01	0.01
Fat color^7^	3.20	4.80	−1.60	(−2.9, −0.2)	0.40	0.04	0.02

^1^Body condition score: 1 to 3 = thin; 4 to 6 = normal; 7 to 9 = obese.

^2^Live muscling score: 1 = thinly muscled, 3 = average muscling; and 5 = extremely muscular.

^3^Live weight at university meat lab serving as a covariate.

^4^Marbling scoring: 100 = Practically Devoid^00^; 200 = Traces^00^; 300 = Slight^00^; 400 = Small^00^; 500 = Modest^00^; 600 = Moderate^00^.

^5^Maturity scoring system: 100 = A^00^; 200 = B^00^; 300 = C^00^; 400 = D^00^; 500 = E^00^.

^6^Carcass muscle scoring system: 1– = 1; 1○ = 2; 1+ = 3; 2– = 4; 2○ = 5; 2+ = 6; 3– = 7; 3○ = 8; 3+ = 9.

^7^Fat color: 1 = white; 2 = creamy white; 3 = slightly yellow; 4 = moderately yellow; 5 = yellow; 6 = neon yellow.

Hot carcass weight was 20.10 kg greater (*P* = 0.05), carcass dressing percentage was 3.94 percentage units greater, and cold carcass weight was 20.4 kg greater (*P* = 0.05) for boner cows compared with leaner cows, whereas cooler shrinkage was not different (*P* = 0.26) between boner and leaner cows. Backfat thickness was 0.39 cm greater (*P* = 0.03), and ribeye area was 9.17 cm^2^ (*P* = 0.05) greater for boner cows compared with leaner cows, whereas KPH fat was not different (*P* = 0.07) between boner and leaner cows. Overall, USDA yield grade was not different (*P* = 0.72) between boner and leaner cows. Marbling score was greater (*P* = 0.05) in boner cows compared with leaner cows with a magnitude of 1.31 marbling score units (boner cows = Small^44^ and leaner cows = Slight^13^). Skeletal maturity was younger (*P* = 0.02) in boner cows compared with leaner cows with a magnitude of 1.21 skeletal maturity units (boner cows = D^66^ and leaner cows = E^88^). Similarly, boner cows had younger lean maturity scores compared to leaner cows; however, these values were not different (*P* = 0.26). Carcass muscle score was different (*P* = 0.01) between boner cows and leaner cows, with scores of 6.0 and 3.8, respectively. Fat color scores were 1.6 lower (i.e., whiter; *P* = 0.02) for boner cows compared with leaner cows (boner cows = 3.2; leaner cows = 4.8).

### Carcass Cutting Yields

The side weights of boner cows were 9.8 kg heavier than those of leaner cows (Table 2). In all instances, boner cows yielded subprimals that were greater in weight than leaner cows, yet only a few of these were statistically different in this study [likely due to the small sample sizes (n = 10 cows/classification) and variation between carcasses]. The inside round (*P* = 0.01), outside round (*P* < 0.01), strip loin (*P* = 0.04), top sirloin (*P *= 0.04), and flank (*P* = 0.03) were all greater in boner cows than in leaner cows. However, there were a few instances in which the subprimal weights for leaner cows had a greater percentage of the side weight. Of importance, numerically, the knuckle, bottom sirloin flap, tenderloin, ribeye roll, and chuck tender were all greater when expressed as a percentage of the side weights for leaner cows. Additionally, leaner cows had a greater amount of bone, both on a weight basis (*P* = 0.05) and on the percentage of the side weight basis (*P* = 0.01), when compared to boner cows.

**Table 2.** A comparison of carcass-cutting yields between boner and leaner beef cull cows^1^

Variable	Boner	Leaner	Effect (Boner—Leaner)	95% CI	SED	Covariate *P* value	*P* value
Observations, n	10	10					
Side weight, kg	131.0	121.2	9.8	(−0.6, 20.2)	4.90	<0.01	0.06
Knuckle, kg	3.52	3.31	0.21	(−0.47, 0.88)	0.32	<0.01	0.52
Knuckle, % side weight	2.70	2.75	−0.05	(−0.67, 0.57)	0.29	0.97	0.87
Inside round, kg	7.40	6.82	0.58	(0.13, 1.02)	0.21	<0.01	0.01
Inside round, % side weight	5.72	5.63	0.09	(−0.31, 0.49)	0.19	0.14	0.63
Outside round, kg	4.23	3.39	0.84	(0.33, 1.34)	0.24	<0.01	<0.01
Outside round, % side weight	3.25	2.75	0.50	(0.15, 0.85)	0.16	0.85	0.01
Eye of round, kg	1.81	1.60	0.21	(−0.03, 0.44)	0.11	<0.01	0.08
Eye of round, % side weight	1.39	1.32	0.07	(−0.07, 0.21)	0.07	0.16	0.32
Strip loin, kg	3.48	2.77	0.71	(0.06, 1.37)	0.31	<0.01	0.04
Strip loin, % side weight	2.62	2.21	0.41	(−0.03, 0.86)	0.21	0.02	0.07
Top sirloin, kg	5.04	4.26	0.78	(0.05, 1.52)	0.34	<0.01	0.04
Top sirloin, % side weight	3.83	3.53	0.30	(−0.21, 0.81)	0.24	0.83	0.23
Bottom sirloin flap, kg	1.40	1.40	0.00	(−0.22, 0.21)	0.10	<0.01	0.97
Bottom sirloin flap, % of side weight	1.07	1.16	−0.09	(−0.24, 0.08)	0.07	0.61	0.28
Tenderloin, kg	1.84	1.77	0.07	(−0.19, 0.33)	0.12	<0.01	0.57
Tenderloin, % side weight	1.41	1.47	−0.06	(−0.23, 0.12)	0.08	0.52	0.52
Flank, kg	0.72	0.61	0.11	(0.01, 0.19)	0.04	<0.01	0.03
Flank, % side weight	0.54	0.50	0.04	(−0.02, 0.10)	0.03	0.05	0.17
Ribeye roll, kg	3.44	3.25	0.19	(−0.36, 0.75)	0.26	<0.01	0.46
Ribeye roll, % side weight	2.62	2.63	−0.01	(−0.33, 0.32)	0.15	0.14	0.96
Chuck tender, kg	1.02	0.97	0.05	(−0.13, 0.24)	0.09	<0.01	0.54
Chuck tender, % side weight	0.79	0.80	−0.01	(−0.15, 0.15)	0.07	0.38	0.96
Brisket, kg	3.99	3.51	0.48	(−0.42, 1.39)	0.42	<0.01	0.27
Brisket, % side weight	2.98	2.87	0.11	(−0.42, 0.64)	0.25	0.18	0.66
Sum of merchandised cuts, kg	37.91	33.65	4.26	(0.82, 7.71)	1.62	<0.01	0.02
Sum of merchandised cuts, % side weight	28.93	27.57	1.36	(−0.40, 3.10)	0.82	0.48	0.12
Lean trim, kg	46.20	42.15	4.05	(−0.51, 8.60)	2.14	<0.01	0.08
Lean trim, % side weight	35.50	34.54	0.96	(−1.93, 3.84)	1.35	0.80	0.49
Fat trim, kg	10.94	4.49	6.45	(−0.53, 13.45)	3.28	0.28	0.07
Fat trim, % side weight	7.75	3.33	4.42	(−0.41, 9.24)	2.27	0.50	0.07
Connective tissue, kg	3.37	3.17	0.20	(−2.80, 3.20)	1.41	0.67	0.88
Connective tissue, % side weight	2.77	2.58	0.19	(−2.20, 2.59)	1.22	0.43	0.87
Bone, kg	30.64	33.42	−2.78	(−5.54, −0.02)	1.29	<0.01	0.05
Bone, % side weight	23.52	28.27	−4.75	(−8.24, −1.26)	1.64	0.36	0.01

^1^Live weight at university meat lab serving as a covariate.

### Merchandizing Values

As expected, boner cows were procured at a greater numerical price at the auction market when compared to leaner cows, and for this study the difference in live cost was $144.14 (*P* = 0.14; Table 3). Because of the additional weight of boner cows, drop value (i.e., value of hide and offal components) was numerically greater (*P* = 0.14) for boner cows compared with leaner cows.

**Table 3.** A comparison of merchandizing value between boner and leaner beef cull cows—June 2023 prices.^1^^,^^2^

Variable	Boner	Leaner	Effect (Boner—Leaner)	95% CI	SED	Covariate *P* value^1^	*P* value
Observations, n	10	10					
Live cost, $/cow	1242.39	1098.25	144.14	(−53.66, 341.94)	92.22	0.02	0.14
Drop value, $/cow	164.37	145.30	19.07	(−7.10, 45.24)	12.20	0.02	0.14
Knuckle value, $/carcass	51.39	49.54	1.85	(−8.76, 12.45)	1.85	<0.01	0.71
Inside round value, $/carcass	103.04	95.79	7.25	(0.32, 14.19)	3.23	<0.01	0.04
Outside round value, $/carcass	68.36	57.38	10.98	(2.38, 19.57)	4.01	<0.01	0.02
Eye of round value, $/carcass	26.32	23.88	2.44	(−1.29, 6.18)	1.74	<0.01	0.18
Strip loin value, $/carcass	56.83	46.42	10.40	(−1.44, 22.24)	5.52	<0.01	0.08
Top sirloin value, $/carcass	82.84	72.21	10.63	(−2.32, 23.57)	6.03	<0.01	0.10
Bottom sirloin flap value, $/carcass	29.64	30.04	−0.40	(−5.42, 4.61)	2.34	<0.01	0.87
Tenderloin value, $/carcass	48.50	46.17	2.33	(−5.21, 9.88)	3.52	<0.01	0.52
Flank value, $/carcass	14.06	12.56	1.50	(-0.39, 3.39)	0.88	<0.01	0.11
Ribeye roll value, $/carcass	56.68	53.76	2.92	(−7.26, 13.11)	4.75	<0.01	0.55
Chuck tender value, $/carcass	14.06	14.17	−0.11	(−2.54, 2.33)	1.14	<0.01	0.93
Brisket value, $/carcass	52.36	49.45	2.91	(−9.49, 15.32)	5.78	<0.01	0.62
Total value of merchandised cuts^3^, $/carcass	600.57	553.53	47.04	(−9.18, 103.25)	26.21	<0. 01	0.09
Lean trim value, $/carcass	584.42	540.46	43.96	(−19.91, 107.83)	29.78	<0.01	0.16
Fat trim value, $/carcass	13.21	8.68	4.53	(−5.82, 14.88)	4.82	0.04	0.36
Bone value, $/carcass	110.70	117.68	−6.98	(−17.11, 3.14)	4.72	<0.01	0.16
Total value when merchandised as subprimal cuts + trim + bone^4^, $/carcass	1303.63	—					
Total value when merchandised as trim + bone^5^, $/carcass	–	1104.71					

^1^Live weight at university meat lab serving as a covariate.

^2^Prices were calculated using June 2023 prices sourced from the USDA Carlot Report (2023) and USDA By-Product Drop Value Report (2023): drop value ($0.06/kg live weight); knuckle value ($1.52/kg); inside round value ($1.43/kg); outside round value ($1.71/kg); eye of round value ($1.51/kg); strip loin value ($1.69/kg); top sirloin value ($1.72/kg); bottom sirloin flap value ($2.19/kg); tenderloin value ($2.67/kg); flank value ($2.06/kg); ribeye roll value ($1.69/kg); chuck tender value ($1.48/kg); brisket value ($1.42/kg); lean trim value ($1.31/kg); fat trim value ($0.16/kg); bone value ($0.36/kg).

^3^Total value of merchandised cuts = knuckle value + inside round value + outside round value + eye of round value + strip loin value + top sirloin value + bottom sirloin flap value + tenderloin value + flank value + ribeye roll value + chuck tender value + brisket value.

^4^Total value when merchandised as subprimal cuts + trim + bone = total value of merchandised cuts + lean trim value + fat trim value + bone value.

^5^Total value when merchandised as trim + bone = lean trim value (assuming all subprimal cuts are sold at lean trim value) + fat trim value + bone value.

Assuming that subprimals were of acceptable quality and could have been merchandised for both boner and leaner cows, most subprimals were of greater numerical value for boner cows compared with leaner cows. Those that were statistically different (*P* ≤ 0.05) included the inside round (+$7.25/carcass) and the outside round (+$10.98/carcass). Overall, when cuts were summed together as the total value of merchandised cuts, the numerical difference (*P* = 0.09) between boner and leaner cows was $47.04/carcass.

Although none were statistically different (*P* ≥ 0.16), the numerical difference for value of lean trim was $43.96/carcass, fat trim was $8.68/carcass, and bone was -$6.98/carcass between boner cows and leaner cows, respectively. Finally, when making the assumption that subprimal cuts could be merchandised from boner cows, total processing revenue was calculated as $1303.63 per boner cow; whereas total processing revenue was calculated as $1104.71 per leaner cow when making the assumption that only trim could be merchandised from the leaner cows.

### Tenderness and Postmortem Proteolysis

Temperature decline during the chilling process between boner and leaner cows for the *longissimus lumborum* were only different at the 4-h (*P* = 0.01), 8-h (*P* = 0.03), and 96-h (*P* = 0.04) sample collection times, and all temperatures with the exception of the 96-h temperature were greater in boner cows than leaner cows (Table 4). The temperature for the *psoas major* was similar (*P* ≥ 0.10) between boner and leaner cows through all data collection times.

**Table 4.** A comparison of carcass temperature (°C) and pH decline between boner (*n* = 10) and leaner beef cull cows (*n* = 10)

Variable	Temperature				pH			
	Boner	Leaner	SEM^1^	*P* value^2^	Boner	Leaner	SEM^1^	*P* value^2^
*Longissimus lumborum*								
2 h	28.50	25.80	3.10	0.06	6.54	6.73	0.08	0.06
4 h	18.90	12.70	2.50	0.01	6.16	6.63	0.11	0.01
8 h	8.70	5.50	1.40	0.03	6.02	6.40	0.12	0.04
24 h	2.30	1.90	0.30	0.11	5.67	5.80	0.03	0.01
48 h	2.00	2.00	0.40	0.99	5.61	5.72	0.02	0.01
72 h	2.00	2.10	0.40	0.49	5.61	5.68	0.04	0.04
96 h	2.10	1.90	0.40	0.04	5.62	5.70	0.02	0.06
*Psoas major*								
2 h	31.30	29.80	2.90	0.29	6.01	6.11	0.14	0.38
4 h	17.00	15.70	2.10	0.48	5.76	5.89	0.12	0.29
8 h	7.90	7.00	1.00	0.46	5.67	5.84	0.09	0.17
24 h	1.90	2.40	0.40	0.22	5.63	5.65	0.04	0.52
48 h	1.70	1.90	0.30	0.10	5.63	5.66	0.03	0.57
72 h	1.80	1.90	0.30	0.53	5.65	5.65	0.06	0.94
96 h	1.90	1.90	0.40	0.88	5.65	5.66	0.04	0.87

^1^Standard error of the mean.

^2^ *P* value = considered statistically different if *P* ≤ 0.05.

**Figure 1.**
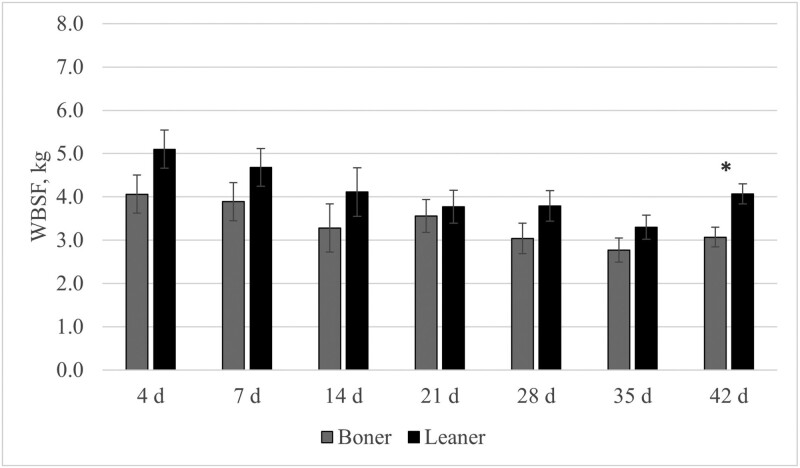
A comparison of Warner–Bratzler shear force (WBSF) of postmortem aging periods of the *longissimus lumborum* between boner and leaner beef cull cows; * indicates a statistical difference *P* ≤ 0.05.

**Figure 2.**
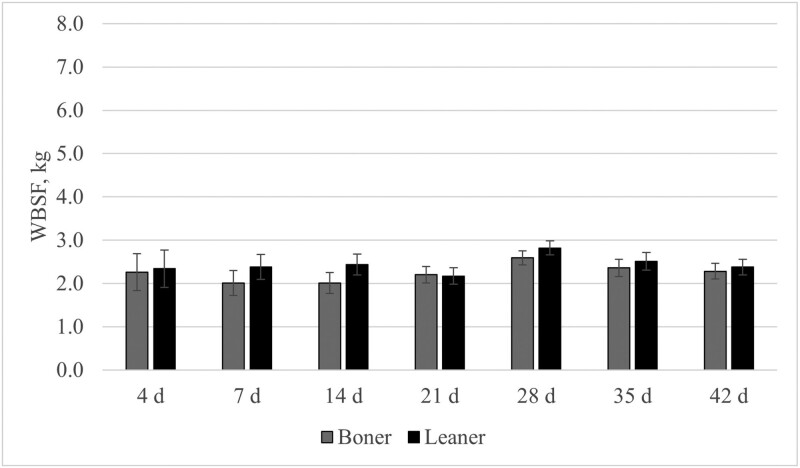
A comparison of Warner–Bratzler shear force (WBSF) of postmortem aging periods of the *psoas major* between boner and leaner beef cull cows; no significant differences were observed for any of the aging times (*P* ≥ 0.15).

The pH decline during the chilling process differed (*P* < 0.05) between boner and leaner cows in the *longissimus lumborum* muscle at 4-h, 8-h, 24-h, 48-h, and 72-h, with the boner cows having lower pH values at each of those time points. The pH for the *psoas major* was similar (*P* ≥ 0.17) between boner and leaner cows through all data collection times. At most aging timepoints, Warner–Bratzler shear force values were similar in both classification groups in both muscle types (Fig. 1 and 2). The only statistical difference noted within these values was at 42 d postmortem in the *longissimus lumborum* muscle (*P* = 0.01). It is worth noting that boner cows had lower numerical values for each aging period throughout both muscles, meaning greater levels of tenderness.

## Discussion

The most recent NBQA – Market Cow and Bull conducted in 2022 (Borders et al., 2024) reported there were more cows evaluated as “too light” muscled than compared to prior audits. In fact, the most recent previous audit conducted in 2016 (Harris et al., 2017) summarized that the highest frequency BCS of beef cows was a BCS = 3. This was a close approximation to what was reported in the current study as the average BCS for the entire population in the present study was 3.45. However, muscling scores postmortem did show greater proportions of lighter muscled cattle as the average score for boner cows was a 2+ and the average for leaner cows was a 1+, neither reaching the “heavy muscled” category (3- or greater). In a previous study, it was the backfat thickness, not the muscle scores or hot carcass weights that were the strongest predictors of lean cutting yields (O’Mara et al., 1998). This was not strongly represented with the cull cows in the present study, particularly with the slight differences for merchandised cuts when expressed as a percentage of each carcass side weight. Body condition score, muscle score, hot carcass weight, backfat thickness, and preliminary yield grade were all seen as significant drivers and thus should all be considered. As seen in Apple et al. (1999), boner cows had the greater percentage of yield, thus generating more revenue for the beef processor. While this pattern did hold true in the current study, it was not a considerable change that would warrant altering production methods. Leaner cows who are of lower BCS come at a more discounted price in the live auction setting, and while not generating as much revenue as boner cows, thus this group could have been seen as more profitable when factoring in live costs.

However, further studies should investigate if feeding leaner cows for a greater period may create more value for producers and limit packer/processor risks such as non-ambulatory animals and carcass and/or meat quality defects. Such packer/processor risks were likely not fully addressed because of the small sample size in this study. For instance, leaner cows are likely weaker and have more challenges navigating trailer ramps and extensive chute systems in large processing plants, challenges cattle in this study were not exposed to. In addition, as cattle age, the amount of myoglobin present increases, creating a darker red color in the lean tissue. The increased myoglobin becomes a potential problem as various studies have shown that consumer perception of product is of the upmost importance, and that cattle further along in their maturity pattern may not be suitable for marketing subprimals in a retail or foodservice setting (Stafford et al., 2022).

The effect of boner versus leaner cows on temperature decline differed at some of the time points measured in the present study, but it is important to note that the *longissimus lumborum* muscle for boner cows was generally at greater temperatures throughout the 24-h sample collection time. This falls within the normal patterns for temperature decline, as fatter cattle (boner group) take a greater length of time to drop in temperature (Aalhaus et al., 2001). There were notable differences for the *longissimus lumborum* muscle as it is covered by subcutaneous fat whereas the *psoas major* hangs freely inside the carcass and is not covered with a layer of fat. The pH decline of the 2 muscle groups demonstrated a relatively normal pattern of decline as the *longissimus lumborum* started at a greater pH and fell to a normal ultimate pH, whereas in the case of the *psoas major*, both classification groups did not see as great of a difference in pH decline from the starting pH to the ultimate pH value. This is because the *longissimus lumborum* is a glycolytic muscle type, which has previously been shown to have a greater decline in pH. Meanwhile, the *psoas major* has a lower starting pH and does not show as much of a decline as it is an oxidative muscle type, and in recent studies, it has been shown to have less of a decline as the muscle type is not as equipped to mobilize the excess of glycogen and potentially stop glycolysis prematurely (Chauhan et al., 2019). The Warner–Bratzler shear force values indicated that the boner cow group had a more tender *longissimus* muscle at days 4, 7, and 14. However, both classification groups were of acceptable tenderness (<4.1 kg) by the 21-d aging period for *longissimus lumborum* steaks (Wheeler et al., 1997). One explanation for this is age, as connective tissue (and specifically collagen crosslinking) increases with age and is not readily degraded through cooking. In a study comparing cull cows and bulls (Holló et al., 2017), the study concluded that age was the greatest indicator of connective tissue, thus having the most impact on tenderness as both groups had the same approximate intramuscular fat.

In summary, boner cows yielded a greater valued carcass considering yield and quality grades, and the total yield of merchandised cuts. However, boner cows sold for a premium price during the live sale valued at $144 more than leaner cows. It is important to consider that the products originating from these cows are marketed differently by beef processors, as both subprimal cuts and trim are normally marketed for boner cows, whereas trim is normally marketed for leaner cows creating a greater difference in revenue from each classification of cows. Additionally, both groups of cows were of “C” or greater skeletal maturity, meaning that these cows would automatically be discounted strictly on age. This provides evidence that selling beef cull cows at younger ages or feeding cull cows prior to marketing (sometimes referred to as a re-alimentation period) may prove beneficial to beef producers selling cattle on a live weight basis. In addition, it benefits beef processors to buy more cull cows classifying as a boner BCS (4 to 6), as these cows would likely generate more revenue than their leaner cow counterparts.

## Abbreviations:

BCS: body condition score
IMPS: Institutional Meat Purchase Specifications
KPH fat: kidney pelvic and heart fat
NBQA: National Beef Quality Audit
USDA: United States Department of Agriculture
